# Exploring antibiotic resistance with chemical tools

**DOI:** 10.1039/d3cc00759f

**Published:** 2023-04-05

**Authors:** Willem A. Velema

**Affiliations:** a Institute for Molecules and Materials, Radboud University Nijmegen, The Netherlands Heyendaalseweg 135 6525 AJ Nijmegen The Netherlands willem.velema@ru.nl

## Abstract

Antibiotic resistance is an enormous problem that is accountable for over a million deaths annually, with numbers expected to significantly increase over the coming decades. Although some of the underlying causes leading up to antibiotic resistance are well understood, many of the molecular processes involved remain elusive. To better appreciate at a molecular level how resistance emerges, customized chemical biology tools can offer a solution. This Feature Article attempts to provide an overview of the wide variety of tools that have been developed over the last decade, by highlighting some of the more illustrative examples. These include the use of fluorescent, photoaffinity and activatable antibiotics and bacterial components to start to unravel the molecular mechanisms involved in resistance. The antibiotic crisis is an eminent global threat and requires the continuous development of creative chemical tools to dissect and ultimately counteract resistance.

## Abstract 2

Antibiotic resistance is an enormous problem that is accountable for over a million deaths annually, with numbers expected to significantly increase. Chemical tools can help to uncover the molecular mechanisms involved in resistance development.

## Graphical abstract

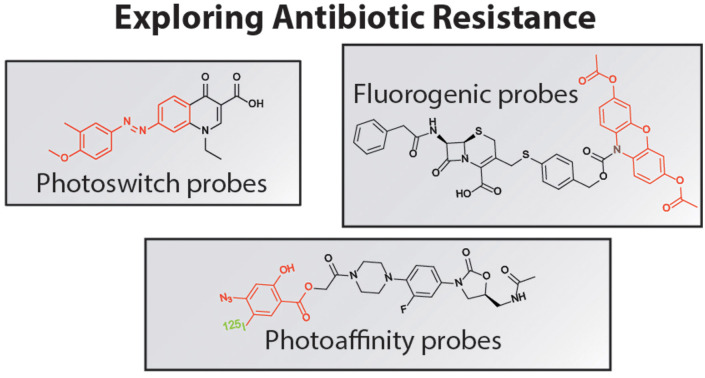

## 1. Introduction

Over the last decades it has become evident that antibacterial resistance is a severe menace to global public health^1,2^ and is likely to impose a large threat to modern medicine.^2^ Increasing reports of truly pan-drug resistant bacteria emphasize the severity and reality of the antibiotic crisis.^3^

The introduction of antibiotics in the twentieth century revolutionized medicine^4^ and allowed clinicians to routinely perform invasive surgery,^5^ treat cancer patients with chemotherapy^6^ and perform risky transplantations, without having to worry about bacterial infections. Dentists can safely perform dental procedures^7^ and veterinarians can treat pets with seemingly innocent infections.^8^ All these advances might be undone if the resistance problem is not adequately tackled.^9^

The societal causes underlying bacterial resistance are well studied.^10^ It is generally believed that resistance emerges due to the (mis)use of large quantities of antimicrobials in human healthcare and animal husbandry, leading to the build-up of enormous amounts of antibiotics in the environment.^11^ This results in a strong evolutionary pressure on bacteria, causing the transfer and capture of resistance genes and the up-regulation of intrinsic resistance mechanisms.^10^ Important mechanisms for resistance include changes in antibiotic influx/efflux and permeability, enzymatic degradation of antibiotics and alteration of antibiotic targets.^12^

Though some of the societal aspects that cause resistance are largely understood, the molecular mechanisms involved can be challenging to study.^12^ This has created a unique opportunity for chemists to leverage their synthetic capabilities and develop tailored chemical tools to dissect the many (bio)molecular mechanisms that underlie antibiotic resistance^13–17^ and to explain antibiotic mechanisms of action. Many innovative chemical approaches have been reported that have led to a plethora of new insights into resistance and how to potentially deal with this global threat.^18^ For example, fluorogenic β-lactam probes are helping to understand β-lactamase activity and its involvement in resistance^19^ while proteomic experiments with photoaffinity labels have helped identify new potential antibacterial targets^20–22^ (*vide infra*).

In this Feature Article, I will discuss some of the most inspiring examples from recent literature together with several of our own contributions to this exciting field. I will first focus on the use of chemically modified antibiotics to study interactions with their bacterial targets and how this has been exploited to study antibiotic mechanisms of action and investigate resistance mechanisms. In the second part of this Feature Article, the application of chemically modified endogenous bacterial small molecules, here referred to as bacterial components, to explore resistance mechanisms is central.

The aim of this Feature Article is not to provide an exhaustive list of all the exciting studies performed, for which I refer to other reviews on the individual topics,^18,19,23–30^ but to highlight various recent chemical approaches to investigate the mechanism of action of antibiotics and how resistance develops. I hope that the examples discussed here will inspire scientists to further expand efforts to counteract the frightening yet real prospect of rising cases of pan-drug resistant bacteria.

## 2. Modified antibiotics as chemical tools

**Fig. 1:**
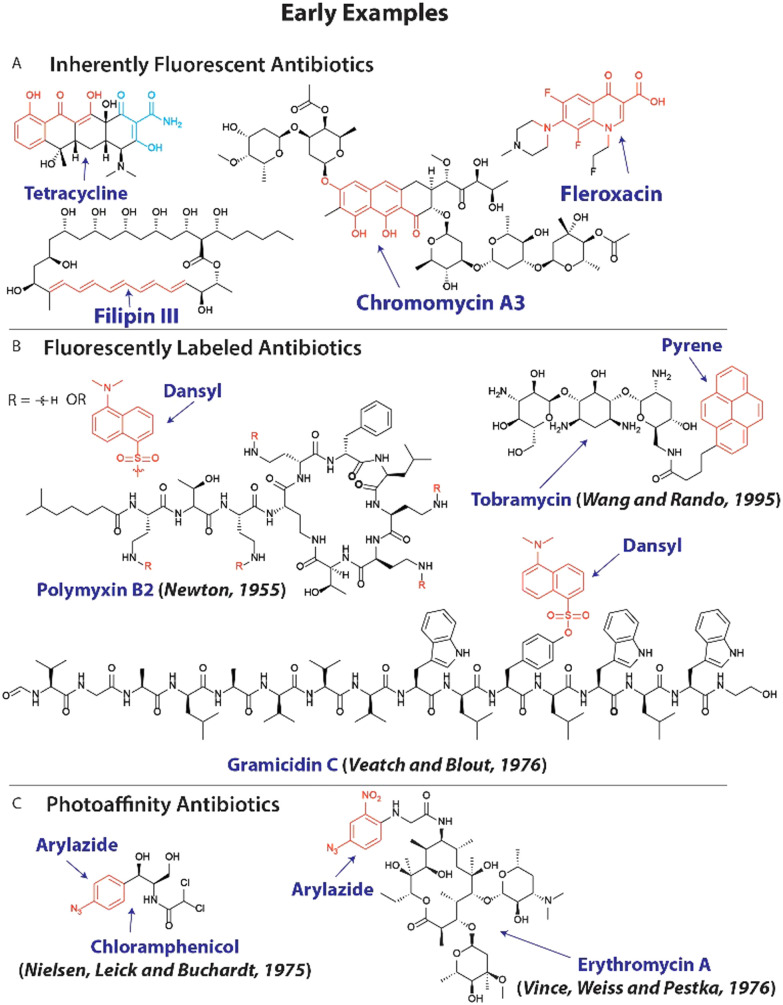
Early examples of modified antibiotics to explore resistance mechanisms. (A) Molecular structure of inherently fluorescent antibiotics, with chromophores displayed in red and cyan. (B) The structure of fluorescently labeled antibiotics with fluorophores shown in red. (C) Examples of antibiotics that are modified with photoaffinity groups (shown in red).

Since many resistance mechanisms involve altered interactions between antibiotics and bacteria, *i.e.* mutated targets and upregulation of antibiotic-inactivating enzymes,^12^ it is sensible to modify existing antibiotics with chemical reporter groups to employ them to probe these altered interactions. Early examples include the use of inherently fluorescent antibiotics like tetracycline^31,32^ to study permeability of intracellular pathogens, chromomycins^33^ to investigate DNA content, antibacterial macrolide polyenes^34^ like filipin III to explore their interaction with lipids and bacterial uptake of fluoroquinolones (Fig. 1(A)).^35^ A variation on this approach entails the chemical modification of existing antibiotics with fluorophores with early studies focusing on polymyxins,^36^ gramicidin C,^37,38^ macrolides^39^ and aminoglycosides (Fig. 1(B)).^40^

Photoreactive groups have been appended to antibiotics to render them crosslinkable to their targets with early examples applied to streptomycin,^41^ puromycin,^42^ macrolides^39^ and chloramphenicol^43,44^ among others (Fig. 1(C)),^45^ and forms the basis for photoaffinity labeling.^46^

More recently, (spatio)temporally activated antibiotics have gathered attention and can be used for temporarily activating antibiotics^23,47^ to dissect their mechanism of action and how bacteria develop resistance against them.^48^

Here, I will discuss these three different types of modifications and highlight several of their successful applications. For a comprehensive overview I refer the reader to recent reviews on the individual topics.^19,23–30^

### 2.1 Fluorogenic antibiotics

**Fig. 2:**
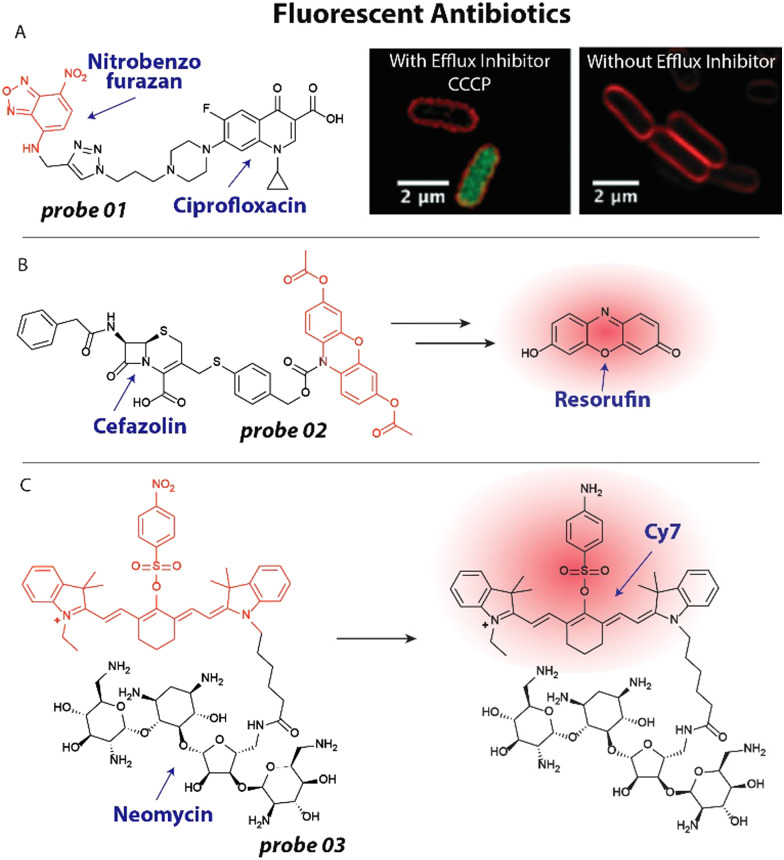
Recent examples of fluorescent antibiotics. (A) Structure of ciprofloxacin modified with a nitrobenzofurazan fluorophore. This probe accumulates in the cytoplasm of *E. coli* (green fluorescence) when treated with the efflux inhibitor CCCP, which is not observed without the efflux inhibitor. Red fluorescence is the membrane dye FM4-64X. Microscopy images are reproduced from ref. 50 with permission from RSC, copyright 2019. (B) Structure of fluorogenic cefazolin probe, which is converted into resorufin upon β-lactamase and esterase activities. (C) A quenched neomycin probe was activated by bacterial nitroreductases and used to image bacterial infections *in vivo*.

The concept of employing fluorescent antibiotics to study their interactions with bacteria has been applied for over half a century.^36,37^ With the looming threat of antibacterial resistance, exploiting fluorescent antibiotics to investigate resistance mechanisms is a viable strategy.^24^ For example, Blaskovich and coworkers have adapted this approach to study efflux mediated resistance mechanisms of several antibiotics.^49–51^ In a recent study,^50^ they appended fluorophores including nitrobenzofurazan (Fig. 2(A)) to the fluoroquinolone ciprofloxacin. The fluorescently labeled ciprofloxacin 01 (Fig. 2(A)) was speculated to be rapidly cleared from the cytoplasm to reduce its activity. When tested on efflux impaired *Escherichia coli* (*E. coli*) a marked 64-fold increase in activity was observed, supporting the hypothesis. To further investigate this, the researchers applied the fluorescent ciprofloxacin 01 to *E. coli* at 50–100 μM and used fluorescent confocal microscopy. Bacteria did not display fluorescence, indicating that the antibiotic was quickly removed from the cytoplasm (Fig. 2(A)). However, when the efflux inhibitor, carbonyl cyanide 3-chlorophenylhydrazone (CCCP) was applied at 10 μM, the cytoplasm displayed bright fluorescence, implying that impaired activity is mostly established through an efflux mechanism.^50^

One particularly attractive and recent strategy entails the use of fluorogenic molecules^52,53^ to study resistance mechanisms and antibiotic mechanism of action. In this case, a fluorophore appended to an antibiotic is temporarily ‘turned off’ and increases in fluorescence upon interaction with its biological target.^52^ This approach has been prominently exploited by Tsien, Rao and others^19,29,54–56^ to investigate β-lactamases that notoriously provide resistance against β-lactam antibiotics including penicillins and cephalosporins. In a recent example that is noteworthy for its simplicity and applicability to urine samples, a cephalosporin was caged with a 3,7-diesterphenoxazine yielding probe 02 (Fig. 2(B)) that released resorufin, a bright fluorophore with a quantum yield of ∼0.75. 3,7-Diesterphenoxazine was attached to the cephalosporin, cefazolin, through a self-immolative linker, that was cleaved upon β-lactamase activity (Fig. 2(B)).^57^ Intracellular esterases hydrolyzed the acetate esters and finally the pro-fluorophore scaffold was oxidized to resorufin, resulting in a ∼1200 fold fluorescent turn-on, only when all triggers were present. Employing probe 02, the authors could detect β-lactam-resistant strains of *E. coli*, *Klebsiella pneumoniae*, *Enterobacter cloacae* and *Serratia marcescens* in urine samples, which could guide clinical decision making and prescription behavior.

Fluorogenic antibiotics have further been applied for *in vivo* monitoring of (resistant) bacterial infections.^58^ Hu and coworkers developed an attractive probe that was effective at imaging infections *in vivo* based on a neomycin analogue with an appended Cy7 fluorophore that was quenched by a *para*-nitro aromatic group to afford probe 03 (Fig. 2(C)).^59^ Nitroreductases within bacterial pathogens reduced the nitro moiety to an amino group, resulting in an ∼8-fold increase in emission at 801 nm. To establish that the probe could be used to image bacterial infections *in vivo* using near infrared (NIR) fluorescence, an infectious mouse model was used. Mice were inoculated with *Staphylococcus aureus* (*S. aureus*) in their hind leg. To investigate selectivity over cancerous tumors, CT26 colon cancer cells were injected in the other hind leg. Mice were injected with 100 μL of 20 μM 03 and scanned using a whole-body fluorescent imager. No detectable signal was observed in the tumor region, while the infection site displayed a ∼2.5-fold background-to-signal fluorescence. The researchers concluded that the probe will likely prove useful to distinguish between bacterial infections, inflammation and cancer, underlining the strength of antibiotic-derived probes to provide selectivity.

### 2.2 Photoaffinity antibiotics

**Fig. 3:**
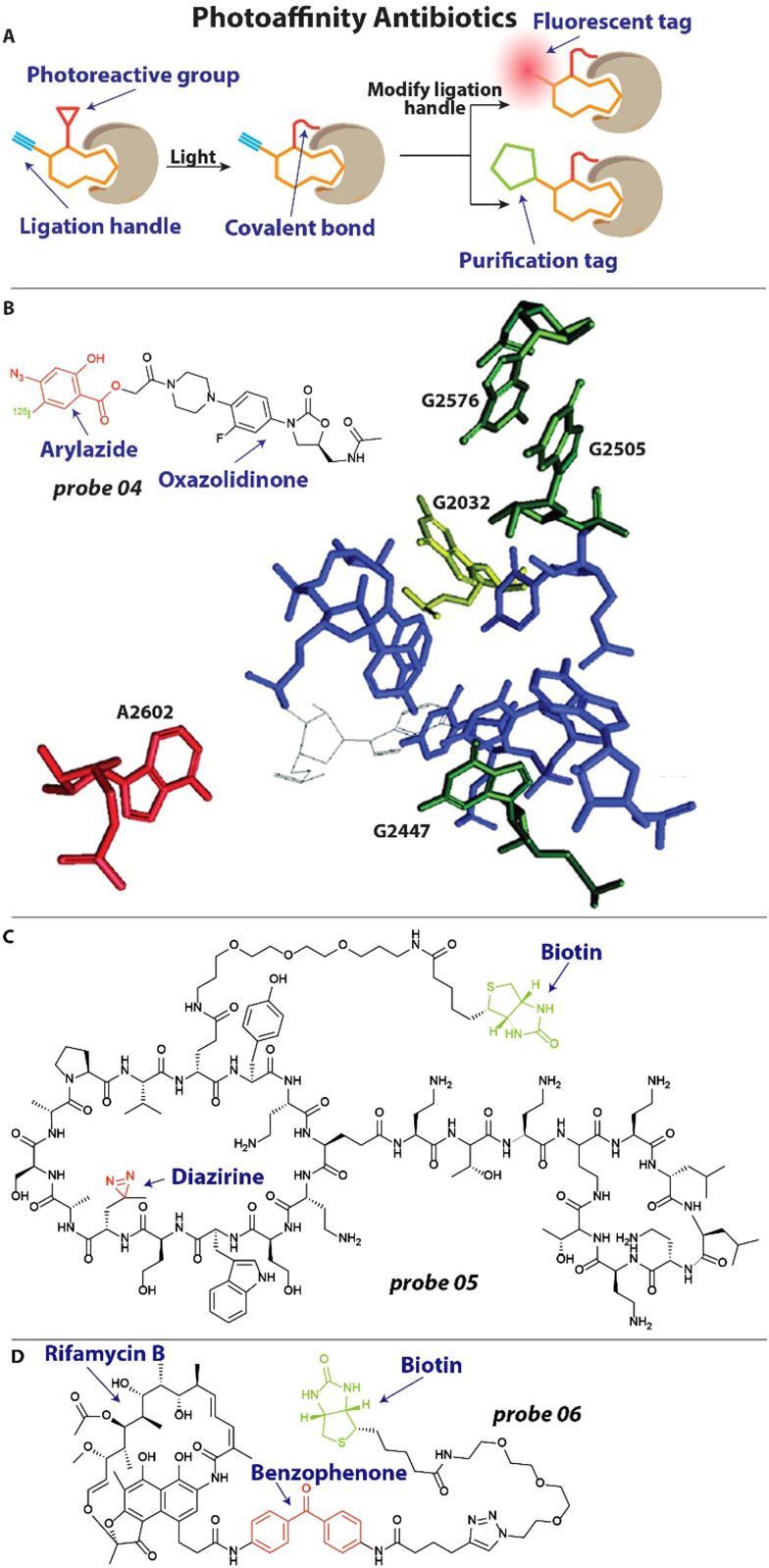
Examples of photoaffinity antibiotics. (A) Schematic illustration of a photoaffinity probe interacting with its biological target. The affinity group is shown in orange, the photoreactive group in red and the ligation handle in blue. (B) Structure of an oxazolidinone modified with an aryl azide photoreactive group and ^125^I radiolabel. This probe revealed the exact interaction site of oxazolidinones within the bacterial ribosome, which labeled nucleotide A2602 (in red). Nucleotides that are involved in resistance when mutated are depicted. Image reproduced from ref. 62 with permission from Elsevier, copyright 2003. (B) Structure of chimeric peptidomimetic photoaffinity probe. A diazirine (red) was included as photoreactive group and biotin (green) as purification handle. (C) A photoaffinity probe based on rifamycin B includes a benzophenone (red) photoreactive group and biotin handle (green) for quantification.

To better understand the mechanism of action of antibiotics and investigate how resistance arises, it is important to explore what cellular components the antibiotic interacts with. A powerful method that is frequently used to this end is photoaffinity labeling.^46^ Antibiotics under investigation can be decorated with a photoreactive group and a reporter or ligation group (Fig. 3(A)). Upon activation of the photoreactive moiety, a covalent bond is established between the antibiotic and its interaction partners (Fig. 3(A)).^46^ Subsequent readout through the reporter group or modification of the ligation handle with a fluorophore or biotin group allows for analysis of the interaction (Fig. 3(A)).

One of the last antibiotic classes to have been approved are the oxazolidinones, including linezolid.^60^ These compounds were developed in the ‘90s and finally approved for use in the 2000's.^61^ It was known that they exert their antibacterial properties by inhibiting protein synthesis, but the exact mechanism and site of interaction with the ribosome remained elusive.^61^ To address this, Colca and coworkers developed an oxazolidinone photoaffinity analogue 04 (Fig. 3(B)), that could crosslink to its target through an aryl azide group and could be quantified with an ^125^I radiolabel.^62^ Bacteria were incubated with 1–2 μM probe 04 and exposed to 254 nm for 2 minutes to effectuate crosslinking. After isolation of ribosomal RNA (rRNA), RNAse H mapping was performed to pinpoint the exact location of labeling, which appeared to be the universally conserved A2602 (Fig. 3(B)). Interestingly, when the experiment was performed in the presence of the unmodified antibiotic, it outcompeted the probe and a reduced signal was observed, underlining the selectivity of the interaction. Furthermore, when analyzing mutations that result in resistance to oxazolidinones they found that these are all in close proximity to the linezolid-interacting site, including G2032 in *E. coli*, and G2447, G2505 and G2576 in Gram-positive bacteria (Fig. 3(B)). This could be highly useful information when designing new antibacterials that will be less prone to resistance development.

Very recently, a chimeric peptidomimetic has been described^63^ that is active against all ESKAPE pathogens.^64^ The molecular scaffold was based on the structure of murepavadin, a macrocyclic β-hairpin, and peptide macrocycle polymyxin and these resulting chimeras displayed excellent activity against ESKAPE pathogens with Minimal Inhibitory Concentrations (MIC) in the range of 0.06–0.25 mg L^−1^. To investigate the mechanism of action, photoaffinity probe 05 (Fig. 3(C)) was prepared that contained a diazirine photoreactive group and biotin enrichment handle.^63^ To ensure that the activity profile remained unchanged, MIC studies were conducted with probe 05 and values of 0.1 mg L^−1^ were found against *E. coli*, similar to the parent compound. Cells were then incubated with the photoaffinity probe and exposed to 350 nm light for 30 minutes to effectuate crosslinking. Using the biotin handle, proteins were enriched with a Streptavidin resin. Captured proteins were digested and analyzed with a mass spectrometer, which lead to the identification of BamA, BamD and LamB as interaction partners. The Bam proteins are essential components of the BAM foldase complex that is responsible for the folding and insertion of β-barrel proteins into the outer membrane of Gram-negative bacteria.^65^ From this and additional *in vitro* experimentation, it was concluded that the interaction of the chimeric peptidomimetic with BAM proteins are responsible for the antibiotic effect.^63^

In another recent example, Wright and coworkers investigated rifamycin resistance using a photoaffinity strategy.^66^ Rifamycin antibiotics exert their activity by inhibiting RNA polymerase (RNAP) but suffer from a high frequency of resistance, which is established through diverse mechanisms. One of which is inactivation through enzymes that are under the control of a 19-bp palindromic sequence termed the rifamycin-associated element (RAE).^67^ Interestingly, it was found that this RAE was also present upstream of several putative helicases^67^ and the researchers hypothesized that these might be involved in rifamycin resistance. To further investigate the mechanism by which these helicases mediate resistance, a photoaffinity probe 06 (Fig. 3(D)) was synthesized based on the rifamycin B scaffold. A benzophenone photoreactive group was appended to secure labeling and a biotin moiety was included for Streptavidin Horseradish peroxidase (HRP) quantification. When incubated *in vitro*, probe 06 readily labeled RNAP after light activation. Interestingly, it was found that the suspect helicase decreases labeling of RNAP by probe 06, indicating that this enzyme can displace RNAP-bound rifamycins and counteract their effect.^66^

These studies illustrate the power of photoaffinity probes based on antibiotic scaffolds to both elucidate their mechanism of action and explore resistance mechanisms.

### 2.3 Activatable antibiotics

A relatively new approach to study antibacterial properties is to control its activity in space and time.^68^ This spatiotemporal control^69^ allows for locally and temporarily increasing or decreasing the concentration of antibiotic, which can help to elucidate the mechanism of action and potential resistance mechanisms. Most commonly used approaches rely on the use of photoprotecting groups to temporarily inactivate an antibiotic,^70^ that can be removed by light for reactivation; and photoswitchable groups that can be incorporated in the structure of an antibiotic and can be isomerized by light exposure to switch between different states of activity.^69^ A third approach entails the use of photodynamic therapy (PDT) agents that can be activated by light to generate reactive oxygen species that kill bacteria,^71^ this approach mostly lacks selectivity and its use to investigate resistance mechanisms is therefore limited.

**Fig. 4:**
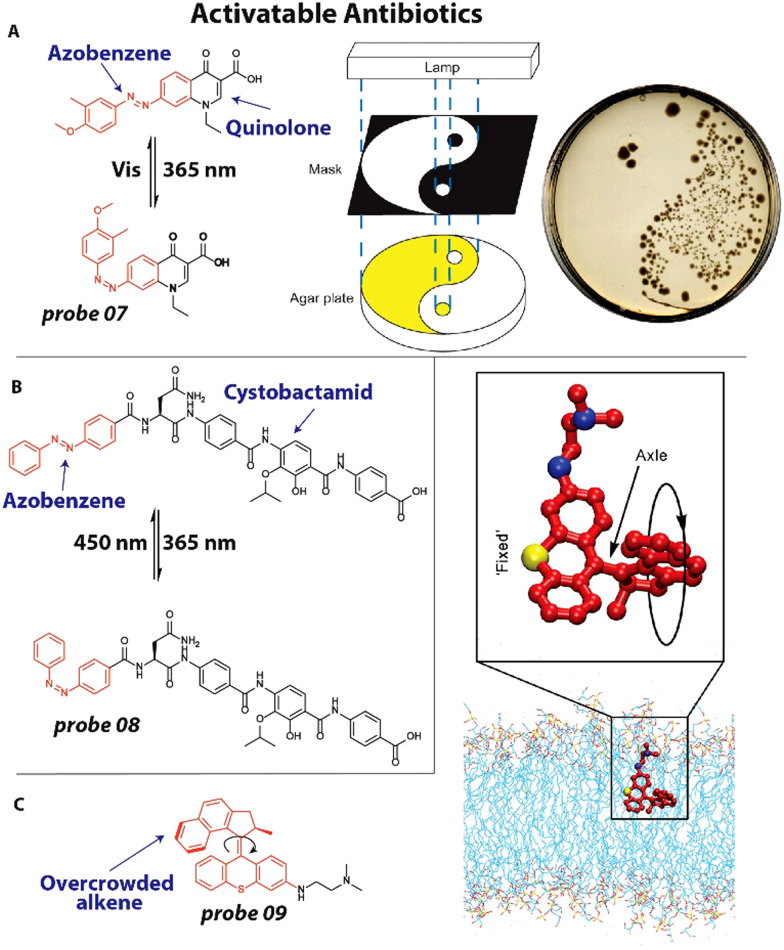
Activatable antibiotics. (A) Structure of azobenzene (red) containing quinolone antibiotic, which can be switched between a *trans* and *cis* isomer with 365 nm and visible light, respectively. Using a mask, bacterial growth could be controlled to predetermined zones on an agar plate. Image reproduced from ref. 47 with permission from Springer Nature, copyright 2013. (B) Structure of photoswitchable cystobactamid 08, with the azobenzene photoswitch displayed in red. (C) Molecular structure of a motor-based antibiotic. A schematic representation depicts a potential mode-of-action of the motor-based antibiotic 09 by incorporating itself in the cell membrane and sequential mechanical disruption. Image reproduced from ref. 72 with permission from AAAS, copyright 2022.

In one of the earliest examples, we appended an azobenzene photoswitch to the molecular scaffold of a quinolone antibiotic.^47^ Azobenzenes can switch between a *trans* and *cis* isomer upon irradiation with light. A small library of nine different compounds were prepared with a varied substitution pattern on the azobenzene and were subjected to MIC studies on *E. coli* CS1562 and *Micrococcus luteus* (*M. luteus*) in both the *trans*- and *cis* isomeric form. In particular compound 07 (Fig. 4(A)) displayed a promising 8-fold difference in activity with a MIC of >64 μg mL^−1^ for the *trans* form and 16 μg mL^−1^ for the *cis* isomer and 16 μg mL^−1^ for the *trans* and 2 μg mL^−1^ for the *cis* isomer on *E. coli* and *M. luteus*, respectively. To demonstrate the spatiotemporal resolution of the photoswitchable quinolone 07, a bacterial patterning experiment was conducted. Compound 07 was dissolved in an agar plate and only part of it was irradiated with light using a mask (Fig. 4(A)) to yield the active *cis* isomer. *E. coli* were inoculated and the plate incubated overnight, resulting in bacterial colonies only at the sections where the antibiotic was not activated because it was covered by the mask.

More recently, Brönstrup and coworkers developed a photoswitchable antibiotic to investigate resistance mechanisms of cystobactamids.^48^ This new class of natural product antibiotics is active against a broad range of pathogens by targeting bacterial gyrase.^48^ This study was one of the first to employ photoswitches to directly investigate resistance. Several resistance mechanisms against cystobactamids have been reported, one of which is binding to AlbA. This protein can bind the antibiotics with nanomolar affinity, which effectively neutralizes its antibacterial properties.^73^ The researchers developed photoswitchable cystobactamid 08 (Fig. 4(B)), which contained an azobenzene photoswitch. To test if the photoswitch could interfere with the resistance mechanism of binding to AlbA, an agar diffusion assay was performed. When compound 08 was used at a concentration of 50 μg mL^−1^*E. coli* growth was fully inhibited in the presence of AlbA. Interestingly, when the antibiotic was isomerized to the *cis*-isomer, no antibiotic activity was observed in the presence of AlbA. This was a particularly striking result, since the *cis*-isomer was found to have higher antibacterial activity than the *trans*-isomer, 0.35 μg mL^−1^*versus* 0.18 μg mL^−1^. This report for the first time employs photoswitches to modulate resistance mechanisms in bacteria and it is expected that this will be extended further to other classes of antibiotics in the coming years.

In a final example, Tour and coworkers developed an activatable antibiotic based on an overcrowded alkene molecular motor.^72^ and is noteworthy for its suspected mechanism of action. They synthesized a library of 19 visible-light activated motors with positively charged groups appended to them that could potentially interact with the negatively charged bacterial membrane. The library was screened against *E. coli* BW25113 while being irradiated with 405 nm light and it was found that in particular fast-rotating molecular motors displayed favorable antimicrobial activity ranging 0.625–32 μM. Conversely, slow-rotating motors did not exhibit antibiotic activity. Compound 09 (Fig. 4(C)), with a tertiary amine appended to the motor, displayed promising antibacterial properties with activity against a broad range of pathogens including *Pseudomonas Aeruginosa (P. aeruginosa)*, *S. Aureus* and *Methicillin Resistant Staphylococcus Aureus* (MRSA). To further explore the mechanism of action of the antibiotic motors, RNA-seq experiments were conducted. Samples treated with 09 and 405 nm irradiation displayed decreased levels of transcripts involved in membrane-associated biological processes as compared to a DMSO control, from which was concluded that the membrane is likely the major target of 09. This was further validated by membrane integrity studies with fluorescent probes, *N*-phenyl-1-naphtylamine and propidium iodide. Up to 2.5-fold increase in fluorescent signal was observed when *E. coli* was treated with the molecular motor antibiotic. From these and other experiments it was concluded that the likely mechanism of action is a mechano-bactericidal effect through physical membrane disruption (Fig. 4(C)). Interestingly, the researchers were unable to isolate resistant mutants, implying that this physical disruption is difficult to counteract by bacteria.^72^

## 3. Modified bacterial components as chemical tools

Apart from modifying antibiotics themselves to render them tools, bacterial metabolites, signaling molecules, and other cellular components (here referred to as bacterial components) that are involved in resistance development can be used as templates for chemical tools as well.^21^ Popular approaches follow a similar trend as compared to modified antibiotic and include the use of fluorescently labeled metabolites, photoaffinity metabolite analogues and activatable bacterial components.

### 3.1 Fluorescent bacterial components

**Fig. 5:**
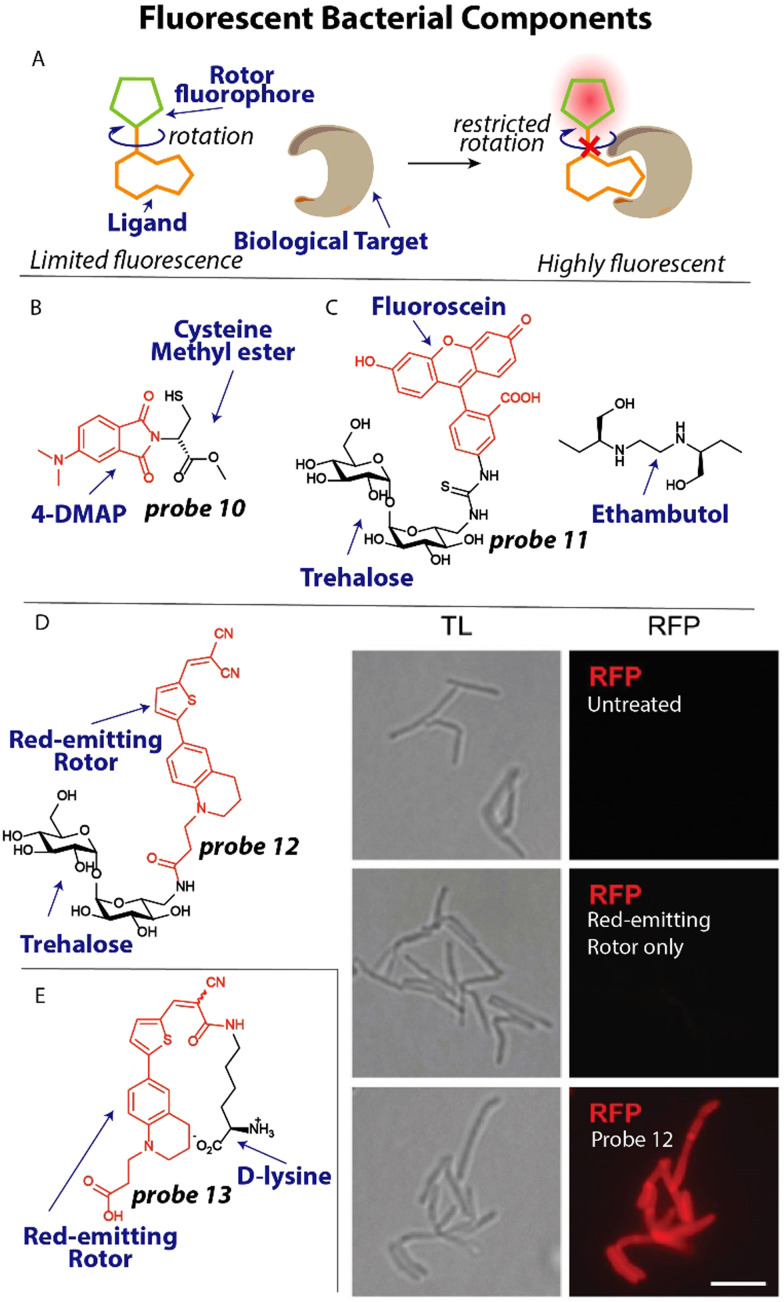
Fluorescent metabolites and bacterial components. (A) Schematic illustration of fluorescent rotor probes. (B) Structure of zinc-binding NDM-reporting probe 10 (C) Structure of trehalose labeled with fluorescein (red) and ethambutol. (D) Trehalose conjugated to a red-emitting fluorophore. When *M. smegmatis*, was treated with this probe they became bright red fluorescent. Image reproduced from ref. 82 with permission from John Wiley and Sons, copyright 2023. (E) Molecular structure of a d-lysine probe that contains a red-emitting rotor and was used to study transpeptidation in bacteria.

To spectroscopically or microscopically visualize the effect of antibiotics on changes in bacterial functioning, it is possible to label metabolites or other bacterial components with a fluorophore or fluorescent rotor (Fig. 5(A)).^74^ One recent elegant example that illustrates this possibility involves the use of an environmentally sensitive dye to detect metallo-β-lactamases. Resistance to many β-lactam antibiotics can be effectuated by New Delhi metallo-β-lactamases (NDM).^75^ These metalloproteins rely on the availability of zinc for their activity. To study metal ion sequestration, Fast, Que and coworkers developed a fluorescent probe that reports on the metalation state of NDM enzymes in bacteria.^76^ Probe 10 (Fig. 5(B)) bears an environmentally sensitive 4-*N*,*N*-dimethylaminopthalimide (4-DMAP) fluorophore that was conjugated to the methyl ester of cysteine that was predicted to bind to zinc in the NDM active site through the thiol group. When 10 μM probe 10 was incubated with the promiscuous NDM-1, a 12-fold increase in fluorescence was observed. When applied in *E. coli*, a clear fluorescent signal was observed around the bacterial periphery using confocal microscopy, in line with the expected location of the NDM enzyme.^77^ Using 1 mM of the β-lactam antibiotic cephalexin in a competition experiment, the observed signal was fully suppressed for a few minutes, after which it quickly recovered which was ascribed to hydrolysis of cephalexin.

The bacterial cell wall and membrane are prominent targets of several antibiotics and many studies have focused on applying fluorescently labeled cell wall components.^78^ Moreover, the composition of the cell membrane can differ significantly between bacterial species and mammalian cells, opening up the possibility of selective detection when targeting these different components. For example, it was found that the disaccharide trehalose is an attractive target to image *Mycobacterium tuberculosis* (*Mtb*) by appending a fluorophore to the trehalose scaffold.^79^ Using this strategy, Bertozzi and coworkers studied mycobacterial membrane dynamics using trehalose probe 11 (Fig. 5(C)).^80^ The mycomembrane is part of the cell envelope that is extraordinarily capable of protecting mycobacteria from antibiotics, rendering *Mtb* infections difficult to treat.^81^ Applying probe 11 at a concentration of 100 μM to *Mycobacterium smegmatis* (*M. smegmatis*), it was found that the tuberculosis drug ethambutol affects the fluidity of the membrane. At concentrations as low as 0.5 μg mL^−1^ ethambutol, the trehalose probe 11 was redistributed across the entire cell surface and finally accumulated at the poles, concluding that ethambutol (Fig. 5(C)) likely acts in part by modulation of the mycomembrane.

One recent example, involves the use of fluorogenic probes to selectively image and detect live *mycobacteria.* To yield trehalose fluorogenic light-up probes,^83,84^ molecular rotors were used that only emit once incorporated into the mycomembrane (Fig. 5(A)).^82^ The advantage is that no wash conditions are required and a light-up signal is indicative of the presence of *mycobacteria* allowing rapid detection. The fluorescent rotor can transition to a twisted internal charge transfer (TICT) state when photoexcited, after which it relaxes through non-radiative relaxation. However, when rotational freedom is limited in a more crowded environment such as the mycomembrane, the TICT state is suppressed resulting in fluorescent emission (Fig. 5(A)). To test this fluorogenic behaviour, compound 12 (Fig. 5(D)) was incubated with *M. smegmatis* at a concentration of 100 μM and immediately imaged without a washing step. A strong fluorescent signal was apparent from the probe treated sample, whereas the untreated and fluorophore only samples did not display any signal (Fig. 5(D)). Using flow cytometry, the signal-to-background was determined when *Mtb* was incubated with probe 12, resulting in an impressive ∼419-fold signal-to-background signal, clearly demonstrating the potential of using this probe for detection of *Mtb*.

The peptidoglycan (PG) cell wall is common to most bacterial species and is a prime target for antibiotics including vancomycin and β-lactams.^85^ Developing selective fluorescent probes that can image peptidoglycan could therefore be useful to study drug resistance against these antibiotics. The PG cell wall consists of glycan strands that are crosslinked by short peptides that contain d-amino acids. Recently, VanNieuwenhze and coworkers reported d-amino acids labeled with fluorescent molecular rotors to study transpeptidation in real-time.^86^

A red-emitting fluorescent rotor was conjugated to d-lysine to afford probe 13 (Fig. 5(E)). Interestingly, when probe 13 was applied in an agarose pad on which *Streptomyces venezuelae* were grown, a clear red signal was observed in growing bacteria using time-lapse microscopy, allowing the visualization of the transpeptidation process. To study the effect of antibiotics on transpeptidation, a high-throughput assay was devised. One mM probe 13 was incubated with the transpeptidase enzyme PBP4 from *S. aureus* and 10 mM synthetic substrates for transpeptidation. A ∼2-fold increase in fluorescence over time was observed, indicative of enzyme activity. The β-lactam antibiotics cefoxitin and carbenicillin fully inhibited the reaction as was apparent from the lack of fluorescence signal, whereas piperacillin, a selective inhibitor for PBP3, displayed no effect on PBP4.

### 3.2 Affinity/activity-based bacterial components

To better understand the molecular processes involved in antibiotic action and resistance and identify important pathways, endogenous metabolites and bacterial components can be labeled with photoaffinity groups to study their biological interactions.^21,87^

**Fig. 6:**
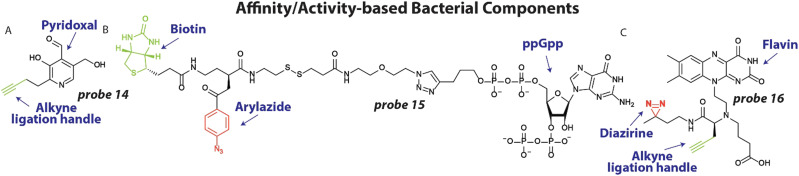
Affinity/activity-based metabolites and bacterial components. (A) Structure of pyridoxal probe 14 with the alkyne ligation handle displayed in green (B) Structure of Magic Spot Nucleotide (MSN) probe 15, with the photoreactive arylazide shown in red and biotin purification handle shown in green. (C) Flavin Mononucleotide (FMN) photoaffinity probe 16 with the diazirine photoreactive group in red and the alkyne ligation handle shown in green.

To expand our arsenal of effective antibiotic drugs to fight resistant bacteria, it is important to identify new potential antibiotic targets.^88^ To this end, Sieber and coworkers set out to uncover pyridoxal phosphate-dependent enzymes that were anticipated to be involved in crucial metabolic processes.^20^ Interestingly, the pyridoxal analogues contain an aldehyde functionality that is essential for its function, which forms a covalent bond with lysine residues of interacting proteins through a Schiff base, foregoing the necessity of a photoreactive group. A small library of pyridoxal-based probes bearing azide or alkyne click handles was prepared. The probes were applied at 100 μM to clinically relevant pathogens including *P. aeruginosa* resulting in 42 enriched proteins after avidin bead enrichment. In particular probe 14 (Fig. 6(A)) enriched many proteins. To decipher if these proteins could be targeted with potential inhibitors, competition experiments were conducted with probe 14 and phenelzine, a non-selective hydrazine-based irreversible inhibitor, in *S. aureus*. The previously uncharacterized protein *A0A0H2XHJ5* that is essential for cell growth was identified and was found to act as a cysteine desulfurase. Importantly, this enzyme could be inhibited by phenelzine with an apparent IC_50_ of ∼14 μM and it was speculated that this contributes to phenelzine's overall antibiotic effect.

In a different study to identify potential antibiotic targets, Jessen and coworkers developed photoaffinity probes of Magic Spot Nucleotides (MSN).^22^ This class of specialized nucleotides are central to the so-called ‘stringent response’ that controls bacterial adaptation to stress, which is important during antibiotic treatment.^89^ To explore the cellular interaction partners of MSN, trifunctional photoaffinity probes were prepared that contain an MSN core, a biotin purification handle and phenylazide photoaffinity group. Probe 15 (Fig. 6(B)) was applied to *E. coli* lysate and irradiated with 310 nm light, to initiate crosslinking. Using streptavidin beads, captured proteins were enriched and analyzed by mass spectrometry. 64 proteins were enriched >4-fold using probe 15, many of which were not known to be interaction partners of MSN. To further validate the results, the authors focused on one enriched enzyme, the phosphatase ApaH, and analyzed if it can process MSN using mass spectrometry. Interestingly, ppGpp on which probe 15 was based, was not converted by ApaH, but pppGpp was. Some of the obtained protein hits may prove to be essential players in the ‘stringent response’ and open up possibilities for antibiotic targeting.

One potential new target for antibiotics are riboswitches. These structured RNA motifs^90^ are usually found 5′ of coding transcripts and can bind small molecule metabolites with high affinity.^91,92^ Upon binding, the RNA structure can change and alter the expression of the coding transcript. Since riboswitches directly control gene expression, they have been speculated as attractive antibiotic targets.^93^ In particular the flavin mononucleotide (FMN) riboswitch is a compelling potential target because it controls essential genes in riboflavin biosynthesis.^94^ Recently, we developed photoaffinity probe 16 (Fig. 6(C)) based on the FMN scaffold, that bears a diazirine photoreactive group and alkyne ligation handle.^95^ Probe 16 was used to measure binding of potential antibiotics to the riboswitch in competition experiments. To demonstrate this, probe 16 was incubated at 10 μM with 2 μM RNA and increasing concentration of the naturally occurring antibiotic roseoflavin that targets the FMN riboswitch. After UV exposure and labeling with fluorescein azide, the amount of labeled RNA was quantified and an IC_50_ value of ∼7.0 μM for roseoflavin was determined. To measure roseoflavin binding in live cells, a similar experiment was performed in *E. coli*. After labeling with probe 16, bacteria were lysed and biotin was appended to labeled RNA using a click reaction. Captured RNA was enriched using streptavidin beads and quantified using RT-qPCR. A clear dose-response curve was obtained with a similar IC_50_ for roseoflavin as was found *in vitro*. It is anticipated that similar approaches can be employed to measure binding of potential antibiotics to RNA targets in live bacteria.

### 3.3 Activatable bacterial components

An attractive approach to investigate molecular processes that are involved in antibiotic activity, bacterial virulence and the emergence of resistance, is to apply chemically modified metabolites and other bacterial components that can be spatially and temporally activated.^23,69^

**Fig. 7:**
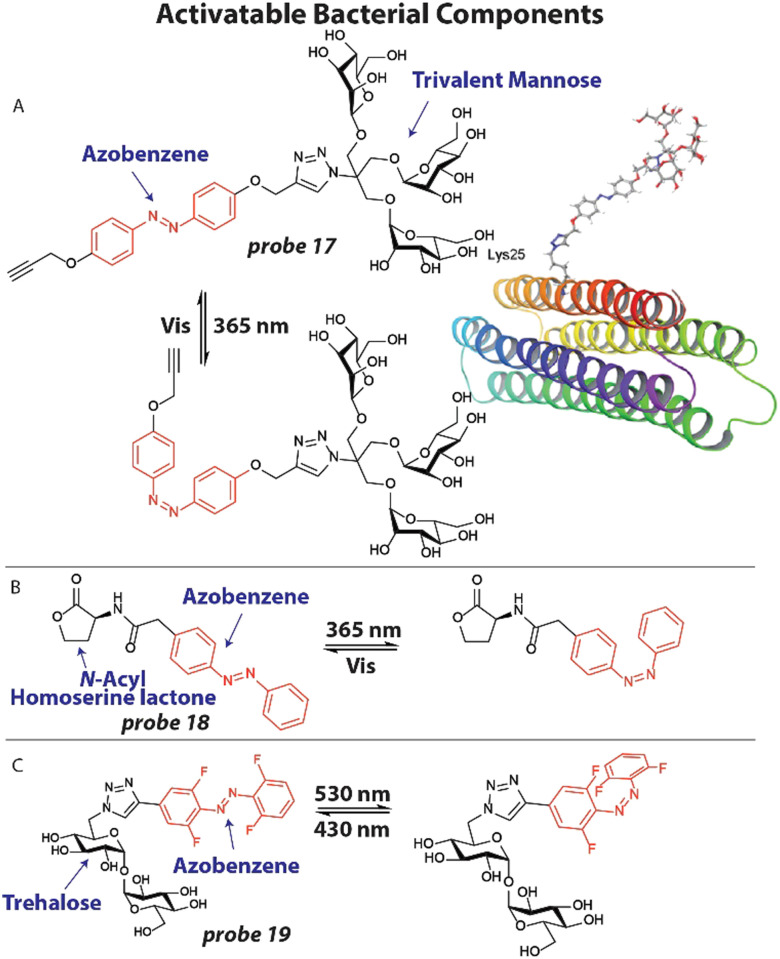
Activatable bacterial components. (A) Structure of photoswitchable mannose ligand 17 with the azobenzene photoswitch displayed in red and the structure of probe 17 when appended to a model glycoprotein. Image reproduced from ref. 97 with permission from John Wiley and Sons, copyright 2019. (B) Structure of photoswitchable quorum sensing autoinducer 18, with the azobenzene shown in red. (C) Molecular structure of photoswitchable trehalose probe 19, with the visible light addressable *ortho*-fluoro azobenzene shown in red.

One example of this strategy involves the application of photoswitchable trivalent mannoside to study bacterial pathogen adhesion to host cells. Host cells are covered by a thick layer of glycoconjugates that can be recognized by bacterial proteins to adhere to the host.^96^ To study this process in detail, Lindhorst and coworkers synthesized photoswitchable mannose ligand 17 (Fig. 7(A)), that can alter its orientation upon light exposure.^97^ Probe 17 was first conjugated to an azido lysine residue of a model glycoprotein that forms a thermodynamically stable four-helix bundle (Fig. 7(A)). Using simulations, the switching trajectories between *trans*17 and *cis*17 when conjugated to the model protein were analyzed. It was found that the trivalent mannose ligand was closer to the protein surface, when in its *cis* configuration with a difference between 2–8 Å compared to the *trans* configuration, depending on the amino acid residue that was used as a reference point. To verify their findings, they applied probe 17 to study bacterial adhesion to human endothelial cells HMEC-1. First, HMEC-1 cells were incubated with an azide-bearing mannosamine for two days to ensure incorporation into glycoproteins on the cell surface. Using click chemistry, probe 17 (200 μM) was attached to the azide groups on the cell surface. GFP-expressing *E. coli* were then incubated with the treated HMEC-1 cells and adhesion was analyzed by fluorescence microscopy. When in the *trans*-form, significant bacterial adhesion was observed, which was normalized to 1.0. However, when probe 17 was switched to its *cis*-isomer by 365 nm light irradiation, adhesion was significantly decreased to ∼0.5, which was at the same level as the negative control. This confirmed the results of the simulations that when in the *cis*-configuration, the trivalent mannose ligand is closer to the surface and likely less available to take part in adhesion.

Bacteria are known to organize themselves and communicate through a process called quorum sensing that allows them to synchronize their gene expression and regulate pathogenicity and antibiotic tolerance.^98^ To accomplish this, bacteria excrete small signaling molecules, called auto-inducers that are recognized by bacterial receptors. We attempted to study and manipulate this process by employing photoswitchable auto-inducers.^99^*N*-Acyl Homoserine Lactones are an important class of autoinducers that contain a lipophilic tail of varying length. To render these compounds photoresponsive, an azobenzene photoswitch was installed in the aliphatic tail (Fig. 7(B)) to afford compound 18. Using modified *E. coli* expressing the Las quorum sensing system^100^ that produces luminescence when activated, compound 18 was tested. Compound 18 effectively activated the Las system in a dose-dependent manner with an EC_50_ ∼25 μM as determined by luminescent signal. When compound 18 was switched to its *cis*-isomer by 365 nm light irradiation an EC_50_ ∼6 μM was found, implying that the *cis*-isomer more effectively activates the quorum sensing system. To further display the potential of probe 18, toxin production that is under the control of quorum sensing was studied. Pyocyanin is produced by *P. aeruginosa* to kill competing bacteria and mammalian cells and has a characteristic absorbance pattern.^101^ Using UV-VIS absorption spectroscopy, the effect of probe 18 on pyocyanin production was investigated. When probe 18 (50 μM, 2×) was incubated with *P. aeruginosa*, only a marginal amount of pyocyanin was measured. However, when probe 18 was switched to the *cis* isomer a significant increase in pyocyanin was observed, estimated at 15 μM. These studies showed that photoswitchable probes can be used to study the natural communication processes between bacteria, which are speculated as antibiotic targets.

In a recent example, Feringa, Szymanski and coworkers developed a photoswitchable trehalose probe 19 that could be addressed with visible light applying *ortho*-fluoro substituents (Fig. 7(C)) and used this to study the mycobacterium membrane.^102^ The trehalose moiety can be recognized by mycobacteria machinery and incorporate it into the cell wall. *M. smegmatis* were grown overnight in the presence of 100 μM 19 and washed. To assess if the probe was metabolically stable, the bacteria were lysed and the lipid fraction was extracted and analyzed by ^19^F NMR. *M. smegmatis* is believed to not contain any fluorinated compounds, so all signals should originate from probe 19.^102^ The untreated control indeed showed no peaks, whereas the experimental signal displayed clear signals. Upon 530 nm light irradiation, the trehalose probe 19 was switched to its predominant *cis* confirmation and clear shifts in signals were observed by ^19^F NMR. Exposure to 430 nm shifted the peaks back to their original values, indicating that photoswitching properties were still fully intact after incorporation into the cell wall. After these early yet exciting experiments, the authors anticipate that improved variants of probe 19 will be subjected to live mycobacteria to control the membrane, which has proven to be a major barrier to antibiotic delivery.^103^

## 4. Conclusions and future perspectives

With the eminent threat of pan-drug resistant bacteria, it is clear that continued research efforts will be necessary to tackle this global problem. The WHO estimates that by 2050 ten million people will die annually from bacterial infections.^104^ Although clear progress is being made to investigate the underlying causes and mechanisms that result in antibacterial resistance, many essential molecular processes remain poorly understood. Chemists now have the opportunity to synthesize sophisticated chemical tools and apply them to answer some of these fundamental questions together with biological scientists. In particular, relatively poorly understood mechanisms warrant further exploration. Examples include antibiotic target protection,^105^ wherein a protein physically associates with an antibiotic target and antibiotic persistence, in which a subpopulation of bacteria is naturally less susceptible to antibiotics,^106^ among others. Chemists are uniquely positioned to help elucidate these processes.

Future research will have to focus on identifying new antibacterial targets to expand our arsenal of effective drugs, while remaining harmless against mammals. Adjuvant^107^ and combination^108^ therapies will likely be necessary to combat severe cases of resistant bacteria. Creative chemical solutions such as hybrid antibiotics might offer a solution.^109^ Lastly, improvements in diagnostic tools will help to identify pathogens at an early stage, opening up doors for more targeted therapies, minimizing the risk for evoking resistance. Chemists will likely be necessary to play a central role in all these scenarios.

## Conflicts of interest

There are no conflicts to declare.

## Supplementary Material
